# Roles of gut microbiota metabolites in the pathogenesis of migraine

**DOI:** 10.3389/fmed.2026.1949484

**Published:** 2026-10-16

**Authors:** Yadi Shi, Yifan Cao, Ziyu Zhang, Ruolin Li, Guangxi Zhou, Peng Xu

**Affiliations:** 1 Jining Medical University, Jining, China; 2 Department of Neurology, Affiliated Hospital of Jining Medical University, Jining, China; 3 Department of Gastroenterology, Affiliated Hospital of Jining Medical University, Jining, China

**Keywords:** gut microbiota, microbiota-gut-brain axis, migraine, short-chain fatty acids, tryptophan

## Abstract

Migraine is one of the most common neurological disorders, with a complex and incompletely understood pathogenesis. Recent studies have implicated the gut-brain axis in migraine pathophysiology. The gut microbiota and microbiota-related metabolites may influence migraine-related processes through neural, immune, endocrine, and metabolic pathways. This review summarizes the potential roles of microbiota-derived metabolites in migraine through the gut-brain axis, with a focus on short-chain fatty acids (SCFAs), tryptophan (Trp)-related metabolites, and nitric oxide (NO)-related metabolic pathways, and their potential effects on neuroimmune signaling, intestinal barrier integrity, and trigeminovascular function. Current evidence suggests that gut dysbiosis and metabolic disturbances are associated with migraine and may modulate neuroinflammatory pathways and central sensitization; however, direct causal evidence in humans remains limited. Interventions targeting the gut microbiota or related metabolic pathways, including probiotics and dietary modifications, may represent potential adjunctive strategies, although current clinical evidence remains limited and heterogeneous.

## 1 Introduction

Migraine is one of the most common neurological disorders, characterized by recurrent moderate-to-severe headache attacks that are often accompanied by nausea, vomiting, photophobia, and phonophobia. Migraine can significantly affect patients' quality of life and social functioning (1). The pathophysiology of migraine is complex and involves multiple interacting mechanisms, including trigeminovascular activation, cortical spreading depolarization (CSD), central sensitization, and calcitonin gene-related peptide (CGRP) signaling (2). In recent years, increasing attention has been paid to the potential association between migraine and gastrointestinal disorders, as well as alterations in gastrointestinal function and the gut microbiota (3, 4).

The gut-brain axis (Figure 1) is a bidirectional communication network connecting the gastrointestinal tract and the central nervous system (CNS) through neural, immune, endocrine, barrier, and metabolic pathways. As an important component of the gut-brain axis, the gut microbiota may influence migraine-related pathophysiological processes by modulating intestinal barrier integrity, immune and inflammatory signaling, neurotransmitter-related pathways, neuropeptide signaling, and microbial metabolite production (5–7). Gut-derived signals may communicate with the CNS through neural pathways, including the vagus nerve, as well as through immune mediators and endocrine signals, and circulating microbial or host-derived metabolites. Conversely, the CNS can regulate gastrointestinal physiology through sympathetic and parasympathetic pathways and the hypothalamic-pituitary-adrenal axis, thereby influencing the intestinal environment and microbial ecosystem (8).

**Figure 1:**
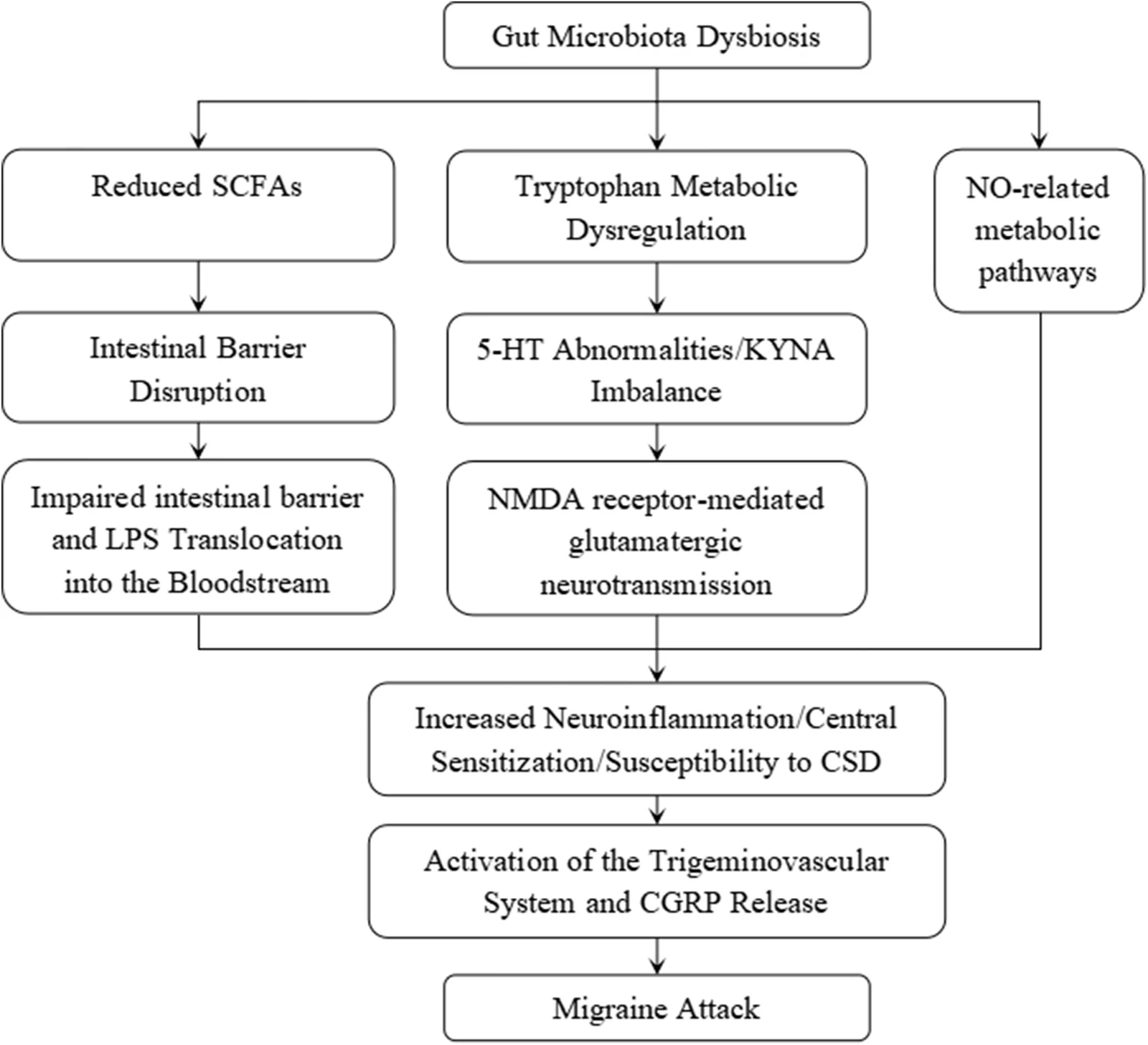
Bidirectional communication within the microbiota–gut–brain axis. The microbiota–gut–brain axis enables bidirectional communication between the gastrointestinal tract and the central nervous system through neural, immune, endocrine, and metabolic pathways. Gut-to-brain signaling involves vagal and enteric neural pathways, immune mediators, gut-associated neuroendocrine signals, and microbial or microbiota-related metabolites, whereas brain-to-gut regulation occurs mainly through the autonomic nervous system, HPA axis, and immune-related mechanisms. Behavioral and psychological factors may further modulate these interactions. The figure is schematic; not all depicted mediators are directly produced by the gut microbiota, and the illustrated pathways should not be interpreted as established causal mechanisms in human migraine. ACTH, adrenocorticotropic hormone; CCK, cholecystokinin; CRH, corticotropin-releasing hormone; GLP-1, glucagon-like peptide-1; HPA, hypothalamic–pituitary–adrenal; IL, interleukin; PYY, peptide YY; SCFAs, short-chain fatty acids; TNF-α, tumor necrosis factor-α; 5-HT, 5-hydroxytryptamine.

Several signaling systems potentially link intestinal and central processes relevant to migraine. These include inflammatory cytokines, CGRP, substance P, vasoactive intestinal peptide, neuropeptide Y, and gut-associated neuroendocrine mediators. Alterations in these pathways, together with gut dysbiosis and impaired intestinal barrier function, may contribute to abnormal gut-brain communication (9). However, most available human microbiome studies are observational, and current evidence does not establish that gut dysbiosis or individual microbial metabolites directly cause migraine. Therefore, associations observed in migraine populations should be distinguished from mechanistic evidence derived from animal models or other neurological and gastrointestinal conditions (7, 10, 11).

This review aims to critically summarize current evidence on the role of gut microbiota and microbiota-related metabolites in migraine pathophysiology, with particular emphasis on short-chain fatty acids, tryptophan-related metabolic pathways, and nitric oxide-related metabolism. We also discuss their potential effects on intestinal barrier function, neuroimmune signaling, CGRP-related pathways, and trigeminovascular nociception, as well as the current evidence for microbiome-targeted interventions. Particular attention is given to distinguishing association from causation and to differentiating human observational and interventional evidence from preclinical and indirect mechanistic findings.

To identify relevant literature, PubMed and Web of Science were searched from database inception to September 2026 using combinations of terms related to four main concepts: migraine or headache, gut microbiota or dysbiosis, microbial or microbiota-related metabolites, and the microbiota-gut-brain axis and its associated mechanisms. Human observational and interventional studies were prioritized, together with migraine-specific preclinical and mechanistic studies. Review articles and studies from other neurological or gastrointestinal conditions were considered when they provided relevant mechanistic context. Studies unrelated to migraine or the microbiota-metabolite-gut-brain axis were excluded from the main evidence synthesis. The reference lists of relevant articles were also screened to identify additional studies (12).

## 2 Evidence linking gut dysbiosis and microbial metabolic alterations to migraine

Existing studies have identified associations between migraine and alterations in gut microbial composition across pediatric, adult, and older populations, as well as in experimental animal models. Compared with healthy controls, individuals with migraine have been reported to show differences in the relative abundance of several microbial taxa and, in some studies, in predicted microbial functions or metabolite-related pathways (Table 1) (13–15). However, most human studies are cross-sectional and therefore do not establish whether gut microbial alterations precede migraine, result from migraine-related dietary or medication changes, or reflect shared environmental and host factors (23, 24).

**Table 1:** Reported gut microbial alterations in migraine and their potential functional relevance.

Gut microorganism	Reported change	Study population/model	Potential functional or metabolic relevance	Evidence level and association with migraine phenotype	Representative author(s)	Country
*Helicobacter pylori*	Higher infection prevalence in some Asian populations	Patients with migraine vs. controls; meta-analysis	Gastrointestinal infection associated with inflammatory and neuroendocrine alterations; not a migraine-specific microbiome signature	Human meta-analysis; associated with migraine status in Asian populations; no consistent association with attack frequency or severity established	Su et al. (16)	China
*Bacteroides*	Increased abundance reported	Children with migraine vs. healthy children	May contribute to microbial metabolic and neuroactive signaling; functional consequences in migraine remain uncertain	Human observational evidence; associated with pediatric migraine status; specific phenotype association not established	Bai et al. (17)	USA
*Parabacteroides*	Increased abundance reported	Children with migraine vs. healthy children	Potentially associated with inflammatory and metabolic functions; migraine-specific functional relevance remains uncertain	Human observational evidence; associated with pediatric migraine status; no specific clinical phenotype association established	Bai et al. (17)	USA
*Eggerthella*	Increased abundance reported	Pediatric and/or adult migraine cohorts vs. controls	Has been associated with inflammatory phenotypes and altered intestinal microbial function	Human observational evidence; reported association with migraine status and, in some cohorts, pain-related clinical measures	Bai et al. (17); Kopchak and Hrytsenko (18); Chen et al. (2, 19, 20)	USA/Ukraine/China
*Clostridium coccoides*	Increased abundance reported	Adults with migraine vs. healthy adults	Associated with carbohydrate fermentation and SCFA-related microbial functions	Human observational evidence; reported association with migraine-related disability in one cohort; causality not established	Kopchak and Hrytsenko (18)	Ukraine
*Candida* spp.	Increased abundance reported	Adults with migraine vs. healthy adults	May reflect altered intestinal microbial ecology and mucosal immune environment	Human observational evidence; associated with migraine status; specific migraine phenotype association not established	Kopchak and Hrytsenko (18)	Ukraine
*Akkermansia muciniphila*	Decreased abundance	Migraine-like mouse model vs. control mice	Associated with intestinal mucus-layer integrity and host metabolic homeostasis	Preclinical animal evidence; clinical migraine phenotype not applicable	Wang et al. (21)	China
Lachnospiraceae	Direction of change varied across studies/models	Migraine-like mice and pediatric migraine cohorts	Many members are involved in fermentation and SCFA production, but functional effects are taxon- and context-dependent	Preclinical and human observational evidence; direction not consistent across studies; no specific clinical phenotype association established	Wang et al. (21); Bai et al. (17)	China/USA
*Faecalibacterium prausnitzii*	Decreased abundance	Older women with migraine vs. matched controls	Major butyrate-associated taxon with potential anti-inflammatory and barrier-supporting functions	Human observational evidence; associated with migraine status; direct association with attack frequency or severity not established	Chen et al. (2, 19, 20)	China
*Bifidobacterium longum*	Reduced abundance reported in human migraine; supplementation examined experimentally	Pediatric migraine cohort and migraine-like animal model	May influence intestinal homeostasis and migraine-relevant neuroimmune/trigeminovascular signaling	Human observational + preclinical intervention evidence; reduced abundance associated with pediatric migraine, while supplementation affected migraine-like signaling in animals; clinical therapeutic relevance remains unestablished	Fan et al. (22)	China
Ruminococcaceae	Decreased abundance reported	Children with migraine vs. healthy children	Includes taxa involved in fiber fermentation and SCFA production	Human observational evidence; associated with pediatric migraine status; specific clinical phenotype association not established	Bai et al. (17)	China

This table summarizes reported changes in gut microbial composition in patients with migraine and migraine-related experimental models, together with the study population or model, potential functional or metabolic relevance, level of evidence, and associations with clinical migraine phenotypes where available. Reported alterations involve taxa that have been linked to inflammatory, metabolic, barrier-related, or neuroactive functions, including *Bacteroides*, *Parabacteroides*, *Eggerthella*, *Bifidobacterium longum*, *Akkermansia muciniphila*, and *Faecalibacterium prausnitzii*. However, the direction and magnitude of these changes vary across populations and experimental models, and most human evidence remains observational. Importantly, taxonomic alterations do not necessarily indicate corresponding changes in metabolite concentrations or metabolic activity. Therefore, the functional implications shown in this table should be interpreted as putative unless supported by direct metabolomic or functional measurements.

Early studies primarily provided indirect evidence for a relationship between gastrointestinal microbial factors and migraine. A 2014 meta-analysis reported a higher prevalence of Helicobacter pylori (H. pylori, HP) infection among patients with migraine in some Asian populations (16). Although this finding suggested a possible link between gastrointestinal microbial exposure and migraine, H. pylori infection should not be regarded as evidence of a migraine-specific intestinal microbiome signature. Subsequent studies shifted from individual infectious agents toward broader characterization of gut microbial communities. In pediatric populations, differences in several taxa, including members of Bacteroidetes and Bifidobacterium, have been reported between children with migraine and healthy controls (17, 25).

Alterations in gut microbial composition have also been reported in adults with episodic and chronic migraine. Differences in taxa such as Alcaligenes spp., Clostridium coccoides, and Eggerthella have been described, although the reproducibility and functional significance of these findings remain uncertain (18). In an age- and body mass index-matched cohort of older women, migraine was associated with enrichment of taxa previously linked to inflammatory phenotypes and with lower abundance of several taxa with putative roles in butyrate production, intestinal homeostasis, and carbohydrate fermentation (20). Similarly, patients with chronic migraine (CM) and medication-overuse headache (MOH) have shown altered microbial profiles associated with circulating inflammatory markers (26). These observations suggest possible links between microbial community structure, host inflammation, and microbial metabolic capacity, but they do not establish a causal relationship (25).

Animal studies provide greater experimental control and support the biological plausibility of microbiota involvement in migraine-related pathways. In a nitroglycerin-induced migraine mouse model, alterations in the gut microbial composition were accompanied by changes in predicted pathways related to bile secretion, steroid hormone biosynthesis, glutathione metabolism, and metabolic processes (21). In another experimental study, Bifidobacterium longum supplementation reduced CGRP expression in the trigeminal ganglion and decreased the number of c-Fos-positive neurons in the trigeminal complex (22). These findings indicate that microbiota manipulation can modulate migraine-related trigeminovascular signaling in experimental models; however, their relevance to human migraine and clinical treatment remains to be established.

Kopchak and Hrytsenko used chromatographic mass spectrometry-based microbial characterization to compare patients with migraine and healthy individuals and reported quantitative differences in several bacterial and fungal markers. Some of these microbial alterations were correlated with headache intensity, attack frequency, migraine-related disability, and psychological status (18). These findings further support an association between gut microbial features and migraine phenotype, but they should not be interpreted as direct evidence of metabolite-mediated causality.

Importantly, taxonomic alterations do not necessarily translate directly into changes in metabolite concentrations. A reduction in putative butyrate-producing bacteria, for example, may suggest reduced community capacity for short-chain fatty acid production, but actual fecal or circulating SCFA levels cannot be inferred from taxonomic changes alone (23, 24). Diet, medication use, age, sex, geographical background, gastrointestinal comorbidities, sample handling, sequencing platforms, and bioinformatic methods may all contribute to heterogeneity across studies (5, 8, 22). Therefore, direct metabolomic measurements and integrated multi-omics approaches are needed to determine whether the microbial changes observed in migraine are accompanied by reproducible alterations in specific metabolites and whether these metabolic changes have mechanistic relevance to migraine.

## 3 Metabolite-mediated communication along the microbiota-gut-brain axis

The microbiota-gut-brain axis is a bidirectional communication network linking the gastrointestinal system and the CNS through neural, endocrine, immune, barrier, and metabolic pathways. Alterations in the gut microbiota may influence intestinal barrier integrity and mucosal immune homeostasis, potentially facilitating the translocation of microbial structural components such as lipopolysaccharide (LPS) and pathogen-associated molecular patterns (PAMPs) into the systemic circulation and amplifying peripheral inflammatory signaling (27–30). These processes provide a plausible route through which intestinal microbial alterations may influence migraine-relevant neuroimmune pathways; however, direct causal evidence in patients with migraine remains limited.

Metabolic signaling represents one of the most relevant components of this communication network. Direct microbial metabolites, including short-chain fatty acids (SCFAs) and several tryptophan-derived indole compounds, can influence epithelial barrier function, immune-cell activity, and neuroimmune signaling (5, 31). In parallel, the gut microbiota may indirectly modulate predominantly host-derived pathways, including serotonin, kynurenine, and nitric oxide-related metabolism, through effects on substrate availability, immune signaling, enteroendocrine function, and microbial enzymatic activity (32, 33). These distinctions are important because not all metabolites involved in gut-brain communication are directly produced by microorganisms.

Neural pathways provide an additional route of gut-brain communication. The vagus nerve can transmit intestinal sensory and metabolic signals to the CNS and may interact with immune and enteroendocrine pathways (5, 7). Although noninvasive vagus nerve stimulation has demonstrated clinical efficacy in selected headache disorders (10), this does not establish that its therapeutic effects are mediated by the gut microbiota. Thus, vagal signaling should currently be viewed as a biologically plausible communication pathway rather than direct evidence of a microbiota-dependent migraine mechanism (5, 7).

Immune and humoral pathways may also connect intestinal microbial activity with migraine-related neurobiology. Increased intestinal permeability and systemic inflammatory signaling may influence blood-brain barrier function, glial activity, trigeminal excitability, and neuropeptide signaling, including CGRP-related pathways (28, 30). However, evidence from other neurological or gastrointestinal disorders should be considered indirect mechanistic support rather than direct evidence for migraine (29, 34). Similarly, associations between microbial alterations and inflammatory biomarkers in patients with migraine do not by themselves establish a causal relationship (35).

Overall, the microbiota-gut-brain axis provides a useful mechanistic framework for understanding how intestinal microbial activity may interact with peripheral and central processes relevant to migraine. Within this framework, microbial metabolites and microbiota-modulated host metabolic pathways may influence intestinal barrier integrity, immune signaling, neurotransmitter-related pathways, and trigeminovascular nociception (5–7). The following sections therefore focus on specific metabolic pathways, including SCFAs, tryptophan-related metabolites and pathways, nitrate/nitrite/nitric oxide metabolism, and other emerging microbiota-related signals, while distinguishing direct migraine evidence from preclinical and indirect mechanistic findings (Figure 2).

**Figure 2:**
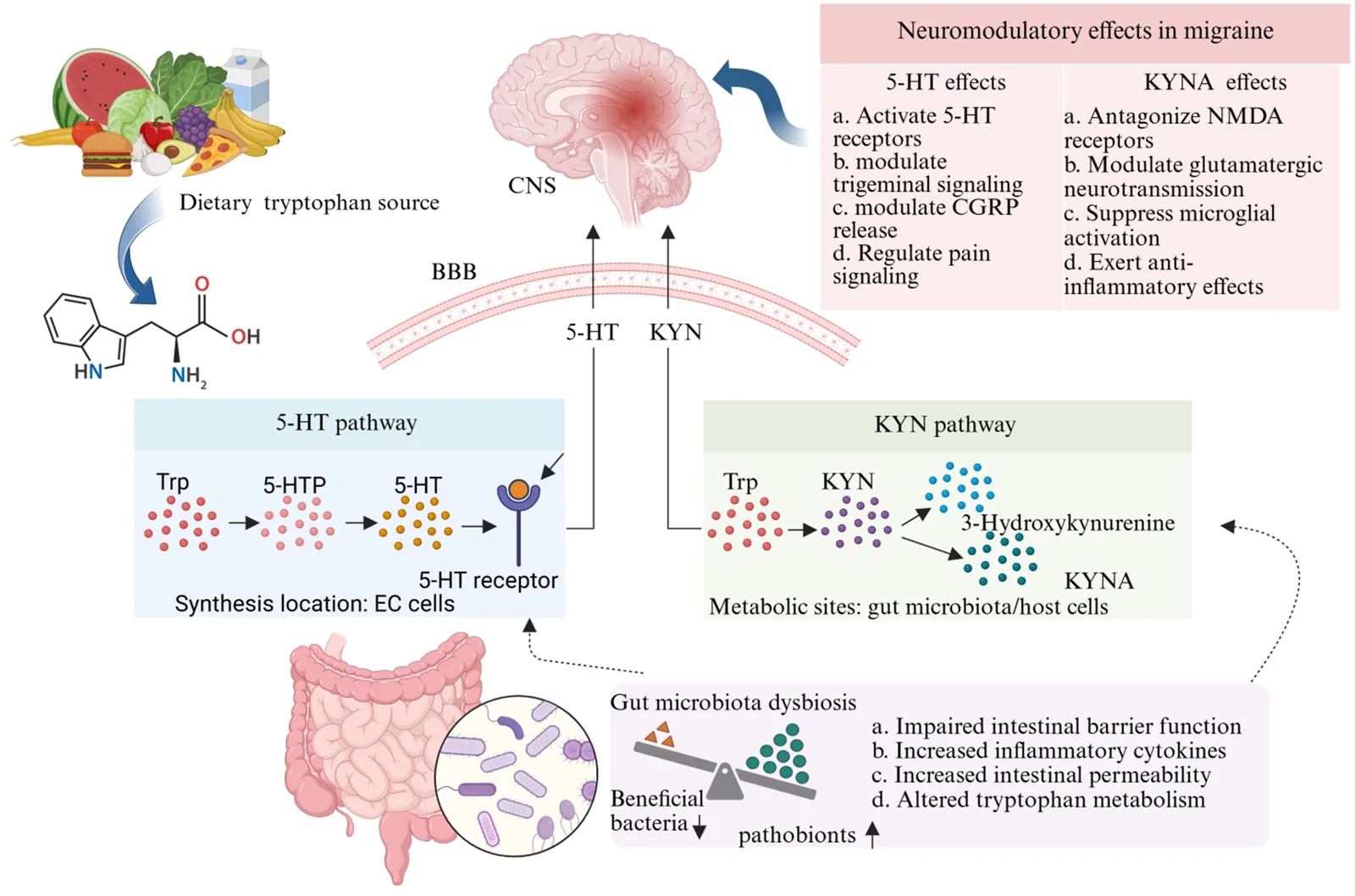
Potential mechanisms linking gut dysbiosis to migraine through the microbiota–gut–brain axis. Gut dysbiosis may impair intestinal barrier integrity and mucosal immune homeostasis, facilitating translocation of microbial components and systemic inflammatory signaling. Altered SCFA signaling and microbiota-modulated neuroactive pathways, together with vagal and HPA-axis signaling, may influence neuroimmune and trigeminovascular processes, including CGRP-related signaling. The depicted pathways are supported by varying levels of preclinical and human observational evidence and should not be interpreted as established causal mechanisms in human migraine. CGRP, calcitonin gene-related peptide; CSD, cortical spreading depolarization; GABA, γ-aminobutyric acid; HPA, hypothalamic–pituitary–adrenal; LPS, lipopolysaccharide; PAMPs, pathogen-associated molecular patterns; SCFAs, short-chain fatty acids; 5-HT, 5-hydroxytryptamine.

## 4 Major microbiota-related metabolites implicated in migraine

Gut microbiota-derived metabolites may influence migraine-relevant biological processes through interconnected immune, metabolic, barrier, and neurovascular pathways. Among the most frequently investigated pathways are SCFAs, Trp metabolites, and NO-related metabolism (Figure 3) (31–33, 36, 37). However, the strength of evidence differs substantially across these pathways, ranging from direct migraine-specific animal studies to human observational findings and indirect mechanistic evidence derived from other neurological or gastrointestinal conditions. The following sections therefore consider each pathway in terms of its microbial contribution, principal host targets, proposed relevance to migraine, and current level of supporting evidence.

**Figure 3:**
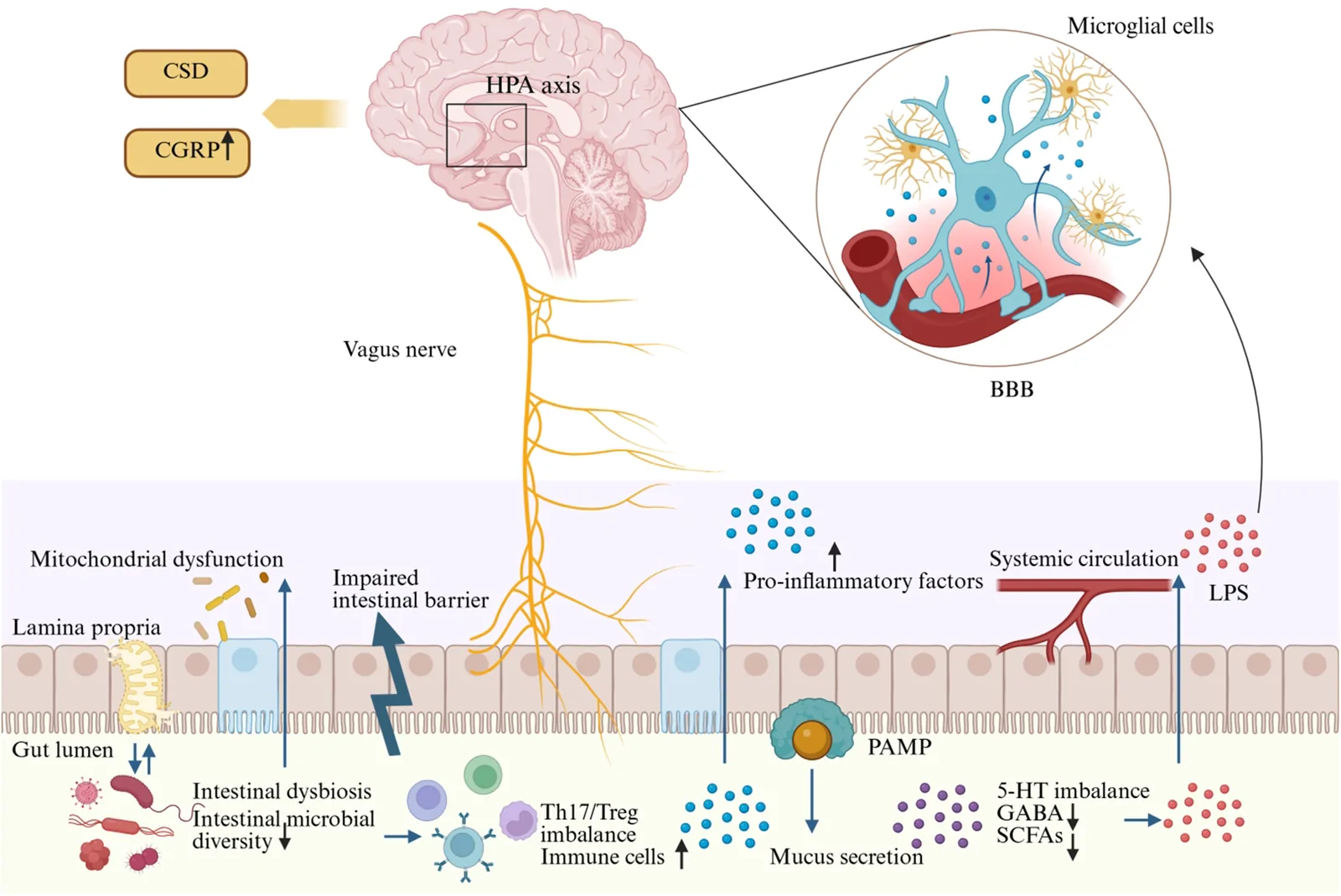
Potential microbiota-related metabolic pathways involved in migraine. Gut dysbiosis may alter short-chain fatty acid (SCFA) production and modulate tryptophan- and nitrate/nitrite/nitric oxide (NO)-related metabolic pathways, with potential effects on intestinal barrier integrity, systemic inflammatory signaling, and neuroactive pathways. Alterations in kynurenine and serotonergic signaling, together with NMDA receptor-related glutamatergic mechanisms, may influence neuroinflammation and trigeminovascular activity, including CGRP-related signaling. These pathways are supported mainly by preclinical and observational evidence and should not be interpreted as established causal mechanisms in human migraine. CGRP, calcitonin gene-related peptide; CSD, cortical spreading depolarization; KYNA, kynurenic acid; LPS, lipopolysaccharide; NMDA, N-methyl-D-aspartate; NO, nitric oxide; SCFAs, short-chain fatty acids.

### 4.1 Short-chain fatty acids

SCFAs are major products of bacterial fermentation of non-digestible dietary carbohydrates and include acetate, propionate, and butyrate. Although these metabolites are often discussed collectively, they differ in abundance, receptor affinity, tissue utilization, and biological effects (31, 33, 36). Their potential relevance to migraine should therefore be considered individually rather than assuming that all SCFAs exert equivalent effects.

#### 4.1.1 Acetate

Acetate is one of the most abundant SCFAs produced in the colon and can enter the systemic circulation. Experimental studies outside the migraine field have shown that microbiota-derived acetate can influence microglial metabolic fitness, maturation, and homeostatic function (38–40). These findings provide a plausible link between microbial metabolism and central neuroimmune regulation; however, they do not constitute direct evidence for a role of acetate in migraine. At present, direct human evidence linking fecal or circulating acetate concentrations with migraine frequency, severity, or treatment response remains limited (36).

Accordingly, acetate should currently be regarded as a biologically plausible neuroimmune mediator rather than an established migraine biomarker or therapeutic target (38, 40).

#### 4.1.2 Propionate and butyrate

Propionate and butyrate exert important effects on intestinal epithelial function, immune regulation, and SCFA-sensitive G protein-coupled receptors, particularly free fatty acid receptor 2 (FFAR2) and free fatty acid receptor 3 (FFAR3) (33, 41). These properties provide several potential mechanisms through which they may influence migraine-related inflammatory and nociceptive pathways.

Migraine-specific preclinical evidence is available from nitroglycerin (NTG)-induced mouse models. Lanza et al. reported that treatment with propionate and butyrate attenuated NTG-induced migraine-like responses, reduced histopathological alterations in the trigeminal nucleus and the expression of pro-inflammatory mediators, improved intestinal mucosal injury, and partially restored abnormalities in enteric neurotransmitter release (42). In subsequent animal experiments, SCFA supplementation also reduced hyperalgesia and pain-related behaviors, ameliorated intestinal tissue damage and permeability abnormalities, and modified gut microbial composition (43). These studies provide direct mechanistic evidence within migraine-like animal models, but they do not establish that endogenous SCFA deficiency is a primary driver of migraine in humans or that SCFA supplementation is clinically effective.

More specific experimental evidence has implicated the SCFA-FFAR2 signaling pathway. In a mouse model involving antibiotic-induced microbiome perturbation, disruption of the gut microbiota prolonged migraine-like pain, whereas microbiota-targeted supplementation restored intestinal acetate and propionate levels and FFAR2 signaling and reduced pain-related behaviors. Importantly, the protective effect was abolished in FFAR2-deficient mice, providing stronger evidence for a causal role of this pathway within the experimental model. In contrast, FFAR3 appeared to be less prominently affected (44). These findings support a functional role for acetate/propionate-FFAR2 signaling in experimentally induced migraine-like pain; however, whether intestinal FFAR2 signaling is similarly altered in patients with migraine remains unknown (41, 44).

#### 4.1.3 Interpretation of the current evidence

Overall, the evidence supporting SCFAs in migraine is strongest at the preclinical mechanistic level. Animal studies indicate that SCFAs may influence intestinal barrier integrity, inflammatory signaling, enteric neurotransmission, and migraine-relevant nociceptive pathways (31, 36, 42–44). Particularly compelling experimental evidence has implicated FFAR2-dependent signaling (44). By contrast, direct human evidence remains limited (45). Taxonomic studies showing reduced abundance of putative butyrate-producing bacteria provide only indirect evidence of altered SCFA metabolism because microbial abundance does not necessarily predict fecal or circulating SCFA concentrations.

Therefore, current data support the biological plausibility of SCFA-related mechanisms in migraine but are insufficient to establish SCFAs as causal mediators, biomarkers, or therapeutic targets in patients (14, 15). Future human studies integrating microbiome profiling with direct measurement of fecal and circulating SCFAs and standardized migraine outcomes are needed to determine their clinical relevance (45).

### 4.2 Tryptophan-related metabolism

Trp is an essential amino acid that is metabolized through three major routes: microbial conversion to indole derivatives, host kynurenine metabolism, and host serotonin synthesis. These pathways differ substantially in their microbial contribution. Indole derivatives are directly produced by intestinal microorganisms (27, 32), whereas the kynurenine and serotonin pathways are predominantly host metabolic pathways that can be indirectly influenced by the gut microbiota through substrate availability, immune signaling, enteroendocrine regulation, and microbial metabolism. This distinction is important when evaluating their potential relevance to migraine (46, 47).

#### 4.2.1 Microbial indole metabolites and AhR signaling

Several intestinal bacteria can convert tryptophan into indole and related metabolites, including indole-3-acetic acid, indole-3-aldehyde, indole-3-propionic acid, and tryptamine. These compounds can act on host receptors such as the aryl hydrocarbon receptor (AhR) and may influence epithelial barrier integrity, mucosal immunity, and neuroimmune signaling (48–50).

Experimental studies in non-migraine neuroinflammatory models have shown that microbiota-derived tryptophan metabolites can modulate astrocyte and immune-cell activity through AhR-dependent mechanisms (49, 50). These findings provide a biologically plausible route linking microbial tryptophan metabolism with central neuroimmune regulation (48). However, direct evidence demonstrating that specific microbial indole metabolites influence migraine pathophysiology in humans remains limited.

Recent migraine-related studies have reported alterations in tryptophan-associated microbial and metabolic signatures, particularly in pediatric populations (25, 51). These findings support further investigation of microbial indole pathways in migraine, but current evidence remains predominantly observational and requires independent replication (45, 52).

#### 4.2.2 Kynurenine pathway

The kynurenine (KYN) pathway is the principal host route of tryptophan catabolism and generates several neuroactive metabolites, including kynurenine, kynurenic acid (KYNA), 3-hydroxykynurenine (3-HK), and downstream products. The gut microbiota may influence this pathway indirectly by altering tryptophan availability, immune activation, and host enzymes involved in tryptophan metabolism. Therefore, KYN metabolites should be considered microbiota-modulated rather than directly microbiota-derived (32, 53, 54).

KYN pathway metabolites can influence glutamatergic neurotransmission, oxidative stress, and immune signaling, all of which are relevant to migraine biology. In particular, KYNA modulates glutamatergic signaling, including NMDA receptor-related mechanisms, whereas 3-HK is a redox-active metabolite associated with oxidative and inflammatory processes (53, 54). However, much of the evidence linking these metabolites to migraine remains either preclinical or observational (55).

Migraine-specific preclinical evidence is available from studies of kynurenic acid analogs. In nitroglycerin-induced migraine models, kynurenic acid analog 1 (KYNA-A1) attenuated hyperalgesia and was associated with changes in neuronal nitric oxide synthase, CGRP-related signaling, and inflammatory mediators (55). These findings support a role for KYN-related signaling in migraine-like nociception within experimental models, but they do not demonstrate that microbiota-dependent modulation of the KYN pathway produces equivalent effects in patients.

Immune signaling may provide an additional connection between the KYN pathway and the microbiota-gut-brain axis. Toll-like receptor signaling and inflammatory pathways can influence host tryptophan metabolism, whereas AhR can respond to several tryptophan-derived ligands (49, 50). Such interactions may be relevant to gastrointestinal and neuroimmune disorders. However, evidence directly establishing a TLR-AhR-KYN pathway as a causal mechanism in human migraine remains insufficient and should be regarded as mechanistic rather than clinical evidence (32, 54).

Clinical studies have also reported alterations in kynurenine-related metabolites in migraine and in functional gastrointestinal disorders (53, 54). Associations involving KYNA, 3-HK, and other pathway intermediates may suggest links between tryptophan metabolism, migraine phenotype, and gastrointestinal comorbidity. However, these findings are largely observational, and the direction of causality remains uncertain (56).

#### 4.2.3 Serotonin pathway

Serotonin (5-HT) is synthesized predominantly by host enterochromaffin cells in the gastrointestinal tract, with additional synthesis in the central nervous system. Although the gut microbiota can influence tryptophan availability and enterochromaffin-cell activity, serotonin should not be considered a purely microbiota-derived metabolite (32, 46, 47).

The role of serotonin in migraine is well established at the neurobiological level. Serotonergic signaling contributes to vascular and nociceptive regulation, and several acute migraine therapies act on 5-HT receptors, including triptans and the selective 5-HT1F receptor agonist lasmiditan (57, 58). However, the clinical efficacy of serotonergic drugs does not by itself demonstrate that gut microbiota-driven changes in serotonin metabolism are responsible for migraine.

Observational studies have suggested associations between dietary tryptophan intake and migraine risk (59), and pediatric studies have reported relationships among gut microbial profiles, tryptophan-related metabolic pathways, and migraine. These findings support a possible microbiota-tryptophan-serotonin connection, but dietary tryptophan intake, peripheral serotonin concentrations, central serotonergic signaling, and microbial tryptophan metabolism represent related but distinct biological processes (45–47, 53). The major relationships among tryptophan, serotonin, and kynurenine metabolism discussed above are summarized in Figure 4.

**Figure 4:**
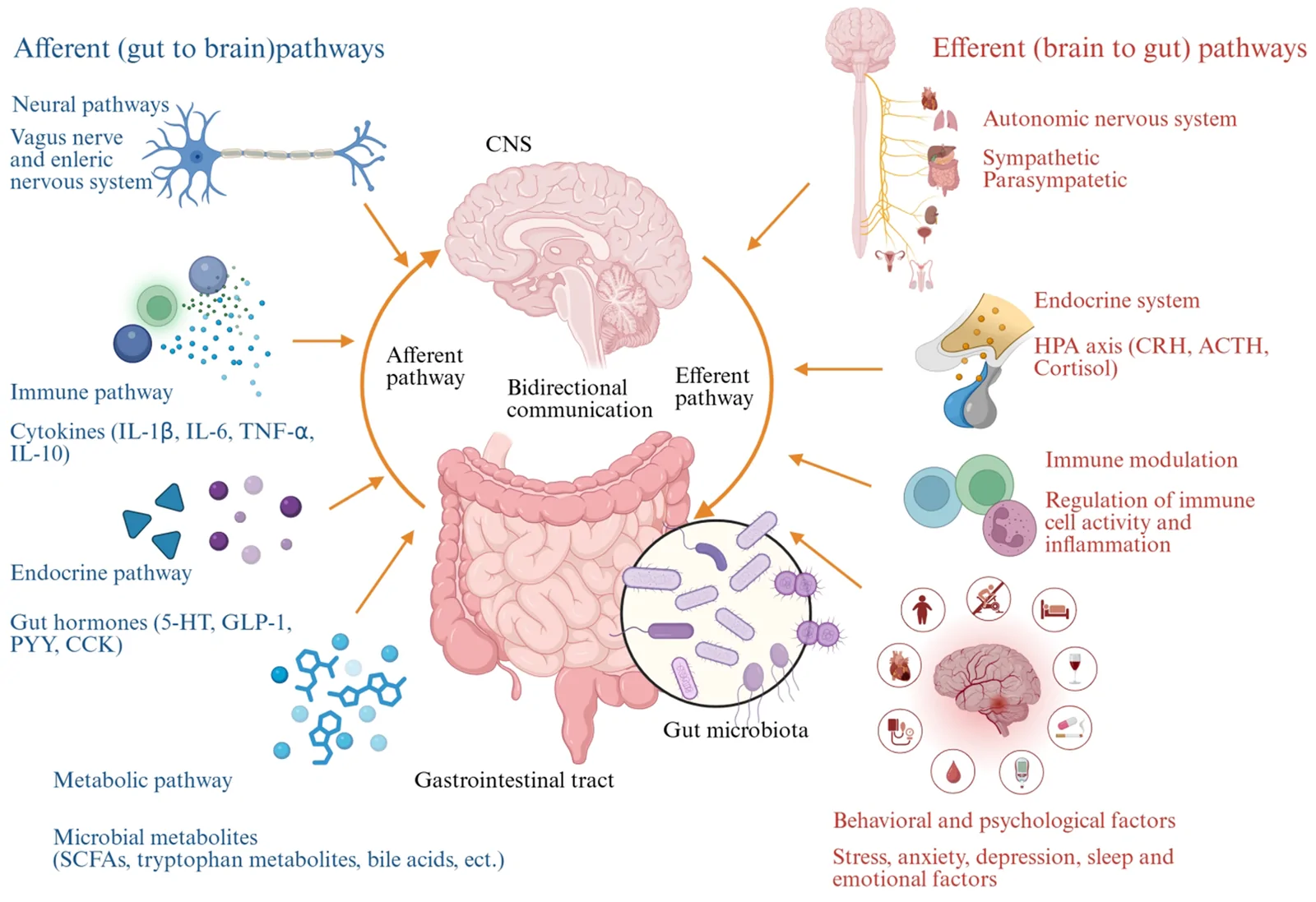
Serotonin and kynurenine pathways in the microbiota–gut–brain axis of migraine. Dietary tryptophan (Trp) is metabolized through several pathways, including host serotonin (5-HT) and kynurenine (KYN) metabolism. In the gastrointestinal tract, enterochromaffin cells convert Trp to 5-HT, which may influence neuroendocrine and nociceptive signaling. The predominantly host-derived KYN pathway generates metabolites including 3-hydroxykynurenine (3-HK) and kynurenic acid (KYNA). KYNA may modulate glutamatergic neurotransmission through NMDA receptor antagonism, whereas 3-HK is associated with oxidative and inflammatory processes. Gut dysbiosis may indirectly influence these pathways by altering Trp availability, intestinal barrier function, and inflammatory signaling. The depicted mechanisms are supported mainly by preclinical and observational evidence and should not be interpreted as established causal pathways in human migraine. Trp, tryptophan; EC cells, enterochromaffin cells; KYN, kynurenine; 5-HTP, 5-hydroxytryptophan; 5-HT, 5-hydroxytryptamine; 3-HK, 3-hydroxykynurenine; KYNA, kynurenic acid; NMDA, N-methyl-D-aspartate; CGRP, calcitonin gene-related peptide.

#### 4.2.4 Interpretation of the current evidence

Overall, evidence linking tryptophan metabolism to migraine is biologically plausible but heterogeneous. Microbial indole metabolites represent the clearest example of directly microbiota-derived tryptophan products, whereas the kynurenine and serotonin pathways are predominantly host pathways that may be indirectly shaped by microbial activity (27, 32). Preclinical studies provide mechanistic support for KYN-related modulation of migraine-like nociception (55), while human studies mainly demonstrate associations rather than causality (52, 56).

Future studies should simultaneously assess gut microbial composition, directly measured tryptophan metabolites, inflammatory and barrier-related biomarkers, and standardized migraine outcomes (45, 52). Such integrated approaches will be necessary to determine whether specific tryptophan-related pathways are causally involved in migraine or merely reflect broader metabolic and inflammatory changes associated with the disorder.

### 4.3 Nitric oxide-related metabolites

Nitric oxide (NO)-related metabolism represents another potential interface between microbial metabolic activity and host neurovascular signaling in migraine. Unlike SCFAs and microbial indole metabolites, however, NO is not exclusively microbiota-derived. Host nitric oxide synthases (NOS), including neuronal NOS (nNOS), endothelial NOS (eNOS), and inducible NOS (iNOS), are major sources of NO, whereas oral and intestinal microorganisms can participate in NO-related metabolism through the nitrate (NO_3_^−^)-nitrite (NO_2_^−^)-NO pathway. This distinction is important because the well-established effects of host-derived NO in migraine should not be directly interpreted as evidence for a microbiota-derived mechanism.

#### 4.3.1 Microbial nitrate-nitrite-NO metabolism

Dietary nitrate enters the enterosalivary nitrate cycle and can be reduced to nitrite by nitrate-reducing microorganisms in the oral cavity, thereby contributing to subsequent NO formation and systemic NO bioavailability. The importance of this microbial pathway is supported by human studies showing that oral microbial composition and nitrate-reduction capacity can modify nitrate metabolism. Rosier et al. demonstrated that individuals with periodontitis had a lower abundance of nitrate-reducing bacteria and reduced oral microbial nitrate-reduction capacity, providing functional evidence that the oral microbiome can influence this metabolic pathway (60). Similarly, Vanhatalo et al. showed in a randomized crossover intervention that dietary nitrate altered the oral microbiome and was associated with changes in systemic NO bioavailability, further supporting a functional link between microbial nitrate metabolism and host NO physiology (61).

The relationship between this pathway and migraine was first suggested by Gonzalez et al., who found higher abundances of microbial genes involved in nitrate, nitrite, and NO reduction in oral samples from individuals with migraine, with a smaller but significant difference also detected in fecal samples (62). More recent evidence has strengthened this association. In a 2025 case-control study including individuals with episodic migraine, chronic migraine, and healthy controls, Cho et al. identified distinct oral microbial signatures in migraine and found that several migraine-associated genera were capable of nitrate and/or nitrite reduction; several of these microbial features were also associated with the number of headache days (63). These findings suggest that altered microbial nitrogen metabolism may be associated with migraine phenotypes.

However, microbial nitrate- or nitrite-reducing capacity should not be equated with increased *in vivo* microbial NO production. The presence of relevant bacterial genes or taxa indicates functional potential rather than actual metabolite flux, and current human studies have not established that microbiota-derived NO or related nitrogen oxides reach the trigeminovascular system at concentrations sufficient to modify migraine attacks. Thus, the available evidence supports an association between microbial nitrate metabolism and migraine rather than a confirmed causal microbiota-derived NO pathway.

#### 4.3.2 NO-related signaling in migraine

Evidence for a role of NO-related signaling in migraine itself is considerably stronger than evidence specifically implicating microbial NO production. NTG, a pharmacological NO donor, can provoke delayed migraine attacks in susceptible individuals and has therefore become an established experimental provocation paradigm. A recent narrative review by Olesen summarized the development of pharmacological migraine provocation models and emphasized that NTG can induce delayed migraine attacks in people with migraine, while also highlighting the distinction between pharmacological provocation and spontaneous migraine pathophysiology (64). This distinction is important because NTG exposure provides evidence that NO-sensitive signaling can engage migraine pathways, but does not by itself demonstrate that an increase in endogenous or microbiota-derived NO is sufficient or necessary for spontaneous migraine.

Preclinical studies from several independent research groups further support the involvement of NO-sensitive signaling in migraine-like nociception. In a chronic intermittent NTG mouse model, He et al. identified protein kinase Cδ as a neuronal mediator of ongoing migraine-like pain; importantly, α-CGRP antagonism reduced the NTG-induced pain phenotype, linking NO-donor exposure to CGRP-related nociceptive signaling (65). Demartini et al. subsequently demonstrated trigeminal hyperalgesia and associated behavioral changes in rats following NTG administration, extending the reproducibility of the model to another laboratory and experimental system (66). In a separate study, Chen et al. found that repeated NTG administration induced neuroinflammation and central sensitization through an IL-17-related mechanism, providing additional evidence that NTG-induced migraine-like phenotypes involve inflammatory as well as neuronal pathways (19).

More recent work has further clarified the complexity of NO-related signaling. Ernstsen et al. demonstrated in a migraine-relevant mouse model that inhibition of NOS activity attenuated NTG-induced hypersensitivity and provided evidence for contributions from iNOS, nNOS, and eNOS, with downstream involvement of peroxynitrite and soluble guanylate cyclase signaling (67). These findings from independent research groups indicate that NO-related migraine signaling is unlikely to depend on a single NOS isoform or downstream pathway. Instead, NO may interact with CGRP signaling, inflammatory responses, oxidative stress, and trigeminovascular nociception.

Importantly, these host-derived mechanisms should be distinguished from microbial nitrate metabolism. Although host-derived NO and reactive nitrogen species may influence intestinal microbial ecology, most evidence for this reciprocal interaction has been obtained outside the migraine field, and its relevance to migraine remains uncertain (68, 69).

#### 4.3.3 Microbiota-NO interactions in migraine

An important step beyond observational microbiome associations is evidence that altering the gut microbiota can modify migraine-like phenotypes. Kang et al. demonstrated that disruption of the gut microbiome altered NTG-induced migraine-related hyperalgesia in mice, whereas microbiome-directed intervention partially restored the phenotype, providing experimental evidence that the gut microbiota can modulate migraine-like nociception (70). Although this study did not establish that microbial NO production was the specific mediator of the observed effect, it provides an important experimental link between the gut microbiome and an NO-donor-based migraine model.

The possibility that microbial nitrate metabolism contributes to this interaction is biologically plausible because oral and intestinal microbial communities possess nitrate- and nitrite-reducing functions. Recent studies have further demonstrated that dietary nitrate can modify the composition of nitrate-reducing oral bacteria, while changes in oral microbial nitrate metabolism can influence systemic NO availability (60, 61, 71). However, whether these microbial changes alter circulating nitrate/nitrite concentrations or NO-related signaling in individuals with migraine has not yet been directly demonstrated.

#### 4.3.4 Interpretation of the current evidence

Overall, the current evidence supports three related but distinct conclusions. First, NO-related signaling has substantial experimental support in migraine, based on human pharmacological provocation studies and animal models involving NOS, CGRP, inflammatory signaling, and oxidative stress (64, 65, 67). Second, oral and intestinal microorganisms possess functional pathways capable of influencing nitrate/nitrite metabolism and systemic NO bioavailability, as demonstrated in human microbiome and dietary nitrate studies (60, 61, 71). Third, migraine has been associated with alterations in nitrate- and nitrite-related microbial signatures, while experimental manipulation of the gut microbiome can modify NTG-induced migraine-like hyperalgesia (62, 63, 70). Nevertheless, these lines of evidence have not yet been integrated sufficiently to demonstrate that microbiota-derived NO or related metabolites constitute a causal mediator of migraine.

The specific contribution of microbiota-mediated nitrate metabolism to migraine remains less clearly established than that of host-derived NO signaling. Future studies integrating microbial functional profiling, dietary nitrate assessment, NO-related metabolites, and standardized migraine phenotypes are needed to determine whether microbial nitrogen metabolism contributes to migraine susceptibility, attack frequency, or chronification.

### 4.4 Emerging microbiota-related metabolites and host-microbial pathways

In addition to SCFAs, Trp-related metabolism, and nitrate/nitrite/NO-related pathways, several emerging microbiota-related metabolites and host-microbial signaling systems may also be relevant to migraine. These include histamine and other biogenic amines (72), intestinal barrier-associated pathways (20, 25, 34, 73), microbiota-endocannabinoidome interactions (74–76), and the estrobolome involved in estrogen metabolism (77–81). Compared with the major pathways described above, migraine-specific evidence for these mechanisms remains more limited and is derived predominantly from observational studies, indirect mechanistic evidence, or experimental models. These pathways should therefore be interpreted according to the source and strength of the available evidence.

#### 4.4.1 Histamine and other biogenic amines

Certain intestinal bacteria possess histidine decarboxylase and can convert dietary histidine into histamine. Microbially produced histamine may influence mucosal immunity, epithelial permeability, vascular tone, mast-cell activity, and sensory signaling, providing a biologically plausible route through which microbial metabolism could interact with headache-related pathways (72, 82).

Histamine itself is relevant to migraine biology. Circulating histamine levels may change during migraine attacks, and exogenous histamine can provoke headache or migraine-like symptoms in susceptible individuals (82). Histamine can also interact with CGRP and other mediators involved in trigeminovascular signaling. However, histamine in the host is also derived from mast cells and other endogenous sources. Therefore, evidence linking histamine to migraine should not be interpreted as direct evidence that bacterial histamine production contributes causally to migraine.

Other microbial amino-acid-derived biogenic amines, including tyramine, tryptamine, and phenethylamine, may also influence vascular, immune, and neurochemical signaling (72). Nevertheless, direct migraine-specific evidence linking microbiota-derived production of these compounds to attack susceptibility or disease chronification remains limited. Future studies combining microbial functional profiling with direct measurement of circulating or intestinal biogenic amines will be necessary to clarify their clinical relevance.

#### 4.4.2 Intestinal barrier-related host-microbial interactions

Intestinal barrier dysfunction may represent an important interface through which microbial alterations influence systemic immune exposure. Barrier-related markers and tight-junction proteins, including zonulin, occludin, and claudins, regulate epithelial integrity and intestinal permeability (34, 35). These molecules are host-derived markers or structural components rather than microbial metabolites and should therefore be distinguished from small-molecule microbial products (20, 25, 73).

In children with migraine, alterations in circulating barrier-related markers have been reported together with gut microbial dysbiosis, changes in tryptophan- and phenylalanine-related metabolic pathways, and mucosal immune activation (25, 45, 51). These findings suggest a possible relationship among microbial composition, metabolic function, and intestinal barrier integrity.

However, current evidence is largely observational (20, 25, 73). It remains unclear whether impaired intestinal barrier function precedes migraine, contributes to migraine pathophysiology, or develops secondarily as a consequence of diet, medication exposure, inflammation, gastrointestinal comorbidity, or other disease-associated factors (23, 24, 34, 35). Longitudinal studies incorporating both barrier biomarkers and direct microbiome/metabolome measurements are therefore required.

#### 4.4.3 The endocannabinoidome and N-acylethanolamines

The endocannabinoid system is involved in nociception, neuroimmune signaling, and trigeminovascular regulation, and alterations in endocannabinoids and related lipid mediators have been reported in migraine (74, 75). The broader endocannabinoidome includes endocannabinoids and structurally related N-acylethanolamines (NAEs), which interact with multiple receptors and metabolic pathways involved in pain and inflammation.

Growing experimental evidence suggests a bidirectional interaction between the gut microbiota and the endocannabinoidome. Changes in microbial composition may influence host lipid signaling, while endocannabinoids and NAEs can in turn affect bacterial growth, physiology, and community structure (76). These interactions provide a potential mechanistic link between microbial ecology and host pain-related lipid signaling.

Nevertheless, current migraine evidence primarily concerns host endocannabinoid biology rather than a demonstrated microbiota-dependent endocannabinoid mechanism (74, 75). Direct evidence that alterations in the gut microbiota-endocannabinoidome axis influence migraine susceptibility, attack frequency, or chronification is still lacking. This pathway should therefore be considered an emerging hypothesis requiring integrated microbiome and lipidomic investigation (76).

#### 4.4.4 Estrobolome and estrogen metabolism

The estrobolome represents another potential interface between the gut microbiota and host hormonal metabolism. It refers to the collection of gut microbial genes involved in estrogen metabolism, including genes encoding enzymes such as β-glucuronidases and β-glucosidases (77, 78). These microbial enzymes can deconjugate estrogen metabolites in the intestinal lumen, thereby facilitating estrogen reactivation and enterohepatic recirculation. In particular, Ervin et al. demonstrated *in vitro* that several human gut microbial β-glucuronidases could reactivate estrone-3-glucuronide and estradiol-17-glucuronide to estrone and estradiol, respectively, providing direct experimental evidence for the enzymatic basis of estrogen reactivation within the estrobolome (79). Recent human data further support this concept, showing that the abundance of gut microbial β-glucuronidase and arylsulfatase genes was associated with deconjugated fecal estrogens and with circulating estrogen metabolites, suggesting a role for gut microbial enzymes in the regulation of estrogen recycling (81).

This pathway may be relevant to migraine because fluctuations in estrogen levels are associated with migraine susceptibility, particularly in relation to the menstrual cycle and other reproductive hormonal transitions (80). Estrogen may also interact with the trigeminovascular system and CGRP-related signaling, providing a potential biological link between hormonal regulation and migraine pathophysiology (80). Therefore, the estrobolome may represent a biologically plausible mechanism through which gut microbial activity could potentially influence hormone-related migraine susceptibility. However, direct evidence linking gut microbial β-glucuronidase activity or alterations in the estrobolome to migraine remains limited. The current evidence should therefore be interpreted as an indirect mechanistic hypothesis rather than as evidence of a microbiota-dependent estrogen mechanism in migraine. Future studies integrating gut microbial functional profiling, microbial β-glucuronidase activity, circulating estrogen metabolites, and well-characterized migraine phenotypes are needed to determine the clinical relevance of this pathway.

#### 4.4.5 Interpretation of the current evidence

Overall, histamine and other biogenic amines, intestinal barrier-related pathways, the endocannabinoidome, and the estrobolome represent biologically plausible extensions of the microbiota-gut-brain framework in migraine (72–82). However, compared with SCFAs and tryptophan-related pathways, the available evidence is less direct and more heterogeneous. In particular, many of the relevant mediators have substantial host-derived sources or reflect microbiota-modulated host pathways and should not be classified simply as microbiota-derived metabolites. Their potential contribution to migraine therefore requires validation through studies that simultaneously assess microbial function, metabolite concentrations, host signaling pathways, and clinical migraine outcomes.

## 5 Therapeutic implications and current clinical evidence

The growing interest in the microbiota-gut-brain axis has stimulated investigation of microbiome-targeted strategies for migraine, including probiotics, synbiotics, dietary modification, and fecal microbiota transplantation (FMT) (83). However, the therapeutic evidence remains substantially weaker than the mechanistic literature, and the clinical relevance of these interventions should be interpreted according to study design and evidentiary strength.

Human randomized trials of probiotics and synbiotics have produced heterogeneous results (83, 84). Some studies have reported reductions in migraine attack frequency, analgesic use, gastrointestinal symptoms, inflammatory markers, or intestinal permeability-related biomarkers following probiotic or synbiotic supplementation (84). In contrast, other placebo-controlled studies have failed to demonstrate significant improvement in primary migraine outcomes (83). Systematic reviews and meta-analyses similarly indicate that the available trials are few in number, relatively small, and methodologically heterogeneous (83).

These findings suggest that probiotics and synbiotics may have potential as adjunctive interventions in selected patients, but the current evidence is insufficient to establish them as standard migraine therapies (83, 84). Moreover, even when clinical improvement is observed, it is often unclear whether the benefit is mediated through changes in microbial composition, specific metabolites, intestinal barrier function, immune signaling, or other mechanisms (83).

Dietary modification represents another potential approach because diet can simultaneously influence migraine triggers, microbial community structure, and substrate availability for microbial metabolism. Observational studies have reported associations between gut-microbiota-oriented dietary patterns and migraine or severe headache (85, 86). However, such studies cannot establish causality, and high-quality dietary intervention trials incorporating microbiome and metabolomic endpoints remain limited.

FMT has also been proposed as a potential strategy for modifying the gut microbial ecosystem in migraine (87). However, controlled human evidence is currently insufficient, and FMT should be regarded as experimental rather than an established treatment for migraine (87). Similarly, direct manipulation of SCFA-related pathways, including SCFA supplementation and FFAR2 signaling, remains primarily preclinical (42–44). Evidence for therapeutic manipulation of tryptophan-related and nitrate/NO-related metabolic pathways in migraine is also currently derived predominantly from preclinical or mechanistic studies (55, 67).

The potential relationship between the microbiota and CGRP-targeted therapies also warrants further investigation. Preclinical evidence suggests that microbiota manipulation can influence CGRP-related signaling in migraine models (22), but there is currently no robust evidence that baseline microbial composition or metabolite profiles predict clinical response to CGRP monoclonal antibodies or other CGRP-targeted treatments.

Overall, microbiome- and metabolite-targeted interventions should currently be considered investigational or adjunctive rather than established migraine therapies. Future randomized controlled trials should incorporate standardized migraine outcomes together with longitudinal microbiome profiling, direct metabolite measurements, dietary and medication data, and relevant inflammatory or barrier biomarkers (14, 15, 88) Such studies will be necessary to determine whether microbiota-related metabolic pathways represent modifiable disease mechanisms, predictive biomarkers, or clinically useful therapeutic targets.

## 6 Summary and outlook

Current evidence supports an association between migraine, alterations in gut microbial composition, and changes in microbiota-related metabolic pathways (14, 15). However, the strength of evidence is heterogeneous. Human studies are predominantly observational and do not establish causality, whereas mechanistic support is derived largely from preclinical models (20, 25). Among the pathways reviewed, SCFAs and microbial indole metabolites represent relatively direct products of microbial metabolism, while kynurenine, serotonin, and nitric oxide-related pathways are predominantly host pathways that may be indirectly modulated by the microbiota (31, 32). These distinctions are important when interpreting their potential contribution to migraine pathophysiology.

Microbiota-related metabolites may influence migraine-relevant processes through effects on intestinal barrier integrity, immune and inflammatory signaling, neuroactive transmitter systems, CGRP-related pathways, and trigeminovascular nociception (31, 54, 72). Nevertheless, direct causal evidence linking individual microbial metabolites to migraine in humans remains limited (14, 15, 45). Similarly, although randomized trials of probiotics and synbiotics have reported potential benefits in selected outcomes, results remain inconsistent, and the available studies are limited by small sample sizes, heterogeneous formulations, differences in treatment duration, dietary and medication confounders, and variability in clinical endpoints (83). At present, probiotics, synbiotics, fecal microbiota transplantation, and direct metabolite-targeted approaches should therefore be considered investigational or adjunctive rather than established treatments for migraine.

Future studies should prioritize longitudinal and well-controlled interventional designs that integrate standardized migraine phenotyping with detailed dietary and medication assessment, metagenomics or metatranscriptomics, and direct measurement of fecal and circulating metabolites (45, 52). Multi-omics approaches combining microbiome profiling, metabolomics, immune and barrier biomarkers, and neuroimaging may help determine whether microbiota-related metabolic signatures precede migraine attacks, reflect disease-associated changes, predict treatment response, or represent modifiable biological mechanisms. Particular attention should be paid to distinguishing directly microbiota-derived metabolites from host metabolic pathways secondarily influenced by the microbiota (14, 15, 45, 52).

Ultimately, the clinical relevance of the microbiota-metabolite-gut-brain axis will depend on demonstrating reproducible metabolic signatures and establishing through prospective and randomized studies that targeted modification of these pathways leads to meaningful improvements in validated migraine outcomes (13). Until such evidence is available, microbiota-related metabolites should be regarded primarily as promising mechanistic and biomarker candidates rather than established therapeutic targets (45, 53).

## References

[1] Eigenbrodt AK Ashina H Khan S Diener H-C Mitsikostas DD Sinclair AJ. Diagnosis and management of migraine in ten steps. Nat Rev Neurol. (2021) 17(8):501–14. 10.1038/s41582-021-00509-5 34145431 PMC8321897

[2] Chen HH Heath KM Patel PR. Migraine. Phys Med Rehabil Clin N Am. (2025) 36(4):701–14. 10.1016/j.pmr.2025.08.002 41167851

[3] Headache Classification Committee of the International Headache Society (IHS). The international classification of headache disorders, 3rd edition. Cephalalgia. (2018) 38(1):1–211. 10.1177/0333102417738202 29368949

[4] Ashina M Katsarava Z Do TP Buse DC Pozo-Rosich P Özge A. Migraine epidemiology and systems of care. Lancet. (2021) 397(10283):1485–95. 10.1016/S0140-6736(20)32160-7 33773613

[5] Ahmed H Leyrolle Q Koistinen V Kärkkäinen O Layé S Delzenne N. Microbiota-derived metabolites as drivers of gut–brain communication. Gut Microbes. (2022) 14(1):2102878. 10.1080/19490976.2022.2102878 35903003 PMC9341364

[6] Ohara TE Hsiao EY. Microbiota-neuroepithelial signalling across the gut-brain axis. Nat Rev Microbiol. (2025) 23(6):371–84. 10.1038/s41579-024-01136-9 39743581

[7] Cryan JF O'Riordan KJ Cowan CSM Sandhu KV Bastiaanssen TFS Boehme M. The microbiota-gut-brain axis. Physiol Rev. (2019) 99(4):1877–2013. 10.1152/physrev.00018.2018 31460832

[8] De Vos WM Tilg H Van Hul M Cani PD. Gut microbiome and health: mechanistic insights. Gut. (2022) 71(5):1020–32. 10.1136/gutjnl-2021-326789 35105664 PMC8995832

[9] Arzani M Jahromi SR Ghorbani Z Vahabizad F Martelletti P Ghaemi A. Gut-brain axis and migraine headache: a comprehensive review. J Headache Pain. (2020) 21(1):15. 10.1186/s10194-020-1078-9 32054443 PMC7020496

[10] Lai Y-H Huang Y-C Huang L-T Chen R-M Chen C. Cervical noninvasive vagus nerve stimulation for migraine and cluster headache: a systematic review and meta-analysis. Neuromodulation. (2020) 23(6):721–31. 10.1111/ner.13122 32166843

[11] Bonaz B Bazin T Pellissier S. The vagus nerve at the interface of the microbiota-gut-brain axis. Front Neurosci. (2018) 12:49. 10.3389/fnins.2018.00049 29467611 PMC5808284

[12] Baethge C Goldbeck-Wood S Mertens S. SANRA—a scale for the quality assessment of narrative review articles. Res Integr Peer Rev. (2019) 4:5. 10.1186/s41073-019-0064-8 30962953 PMC6434870

[13] Qu K Li M-X Gan L Cui Z-T Li J-J Yang R. To analyze the relationship between gut microbiota, metabolites and migraine: a two-sample Mendelian randomization study. Front Microbiol. (2024) 15:1325047. 10.3389/fmicb.2024.1325047 38690367 PMC11058981

[14] Zhang N Tran S Moskatel LS. The gut microbiome and migraine: updates in understanding. Curr Neurol Neurosci Rep. (2025) 25(1):20. 10.1007/s11910-025-01408-8 39985639

[15] Mugo CW Church E Horniblow RD Mollan SP Botfield H Hill LJ. Unravelling the gut-brain connection: a systematic review of migraine and the gut microbiome. J Headache Pain. (2025) 26(1):125. 10.1186/s10194-025-02039-7 40399789 PMC12096802

[16] Su J Zhou XY Zhang GX. Association between helicobacter pylori infection and migraine: a meta-analysis. World J Gastroenterol. (2014) 20(40):14965–72. 10.3748/wjg.v20.i40.14965 25356058 PMC4209561

[17] Bai J Shen N Liu Y. Associations between the gut microbiome and migraines in children aged 7-18 years: an analysis of the American gut project cohort. Pain Manag Nurs. (2023) 24(1):35–43. 10.1016/j.pmn.2022.06.002 35907763

[18] Kopchak O Hrytsenko O. Feature of gut microbiota in patients with migraine and healthy individuals. Georgian Med News. (2022) (327):13–7. 35959927

[19] Chen H Tang X Li J Hu B Yang W Zhan M. IL-17 crosses the blood-brain barrier to trigger neuroinflammation: a novel mechanism in nitroglycerin-induced chronic migraine. J Headache Pain. (2022) 23(1):1. 10.1186/s10194-021-01374-9 34979902 PMC8903553

[20] Chen J Wang Q Wang A Lin Z. Structural and functional characterization of the gut microbiota in elderly women with migraine. Front Cell Infect Microbiol. (2020) 9:470. 10.3389/fcimb.2019.00470 32083024 PMC7001586

[21] Wang D Liu X Shi S Ren T Wang W. Gut microbiota and metabolite variations in a migraine mouse model. Front Cell Infect Microbiol. (2024) 13:1322059. 10.3389/fcimb.2023.1322059 38357211 PMC10864585

[22] Fan PC Chua HH Lin CR Lai TH Chiou LC Lee WT. Targeting the microbiome in pediatric migraine: gastrointestinal manifestations and the therapeutic role of bifidobacterium longum. Gut Microbes. (2025) 18(1):2606487. 10.1080/19490976.2025.2606487 41454664 PMC12758293

[23] Rinninella E Raoul P Cintoni M Franceschi F Miggiano GAD Gasbarrini A. What is the healthy gut microbiota composition? A changing ecosystem across age, environment, diet, and diseases. Microorganisms. (2019) 7(1):14. 10.3390/microorganisms7010014 30634578 PMC6351938

[24] Rothschild D Weissbrod O Barkan E Kurilshikov A Korem T Zeevi D. Environment dominates over host genetics in shaping human gut microbiota. Nature. (2018) 555(7695):210–5. 10.1038/nature25973 29489753

[25] Papetti L Del Chierico F Frattale I Toto F Scanu M Mortera SL. Pediatric migraine is characterized by traits of ecological and metabolic dysbiosis and inflammation. J Headache Pain. (2024) 25(1):171. 10.1186/s10194-024-01871-7 39379796 PMC11462686

[26] Vuralli D Ceren Akgor M Dagidir HG Onat P Yalinay M Sezerman U. Microbiota alterations are related to migraine food triggers and inflammatory markers in chronic migraine patients with medication overuse headache. J Headache Pain. (2024) 25(1):192. 10.1186/s10194-024-01891-3 39516813 PMC11546420

[27] Roager HM Licht TR. Microbial tryptophan catabolites in health and disease. Nat Commun. (2018) 9(1):3294. 10.1038/s41467-018-05470-4 30120222 PMC6098093

[28] Rooks MG Garrett WS. Gut microbiota, metabolites and host immunity. Nat Rev Immunol. (2016) 16(6):341–52. 10.1038/nri.2016.42 27231050 PMC5541232

[29] Levy M Thaiss CA Elinav E. Metabolites: messengers between the microbiota and the immune system. Genes Dev. (2016) 30(14):1589–97. 10.1101/gad.284091.116 27474437 PMC4973288

[30] Braniste V Al-Asmakh M Kowal C Anuar F Abbaspour A Tóth M. The gut microbiota influences blood-brain barrier permeability in mice. Sci Transl Med. (2014) 6(263):263ra158. 10.1126/scitranslmed.3009759 25411471 PMC4396848

[31] Dalile B Van Oudenhove L Vervliet B Verbeke K. The role of short-chain fatty acids in microbiota–gut–brain communication. Nat Rev Gastroenterol Hepatol. (2019) 16(8):461–78. 10.1038/s41575-019-0157-3 31123355

[32] Agus A Planchais J Sokol H. Gut microbiota regulation of tryptophan metabolism in health and disease. Cell Host Microbe. (2018) 23(6):716–24. 10.1016/j.chom.2018.05.003 29902437

[33] Koh A De Vadder F Kovatcheva-Datchary P Bäckhed F. From dietary fiber to host physiology: short-chain fatty acids as key bacterial metabolites. Cell. (2016) 165(6):1332–45. 10.1016/j.cell.2016.05.041 27259147

[34] Fasano A. Zonulin and its regulation of intestinal barrier function: the biological door to inflammation, autoimmunity, and cancer. Physiol Rev. (2011) 91(1):151–75. 10.1152/physrev.00003.2008 21248165

[35] Camilleri M. Leaky gut: mechanisms, measurement and clinical implications in humans. Gut. (2019) 68(8):1516–26. 10.1136/gutjnl-2019-318427 31076401 PMC6790068

[36] O'Riordan KJ Collins MK Moloney GM Knox EG Aburto MR Fülling C. Short chain fatty acids: microbial metabolites for gut-brain axis signalling. Mol Cell Endocrinol. (2022) 546:111572. 10.1016/j.mce.2022.111572 35066114

[37] Karsan N Gosalia H Goadsby PJ. Molecular mechanisms of migraine: nitric oxide synthase and neuropeptides. Int J Mol Sci. (2023) 24(15):11993. 10.3390/ijms241511993 37569369 PMC10418996

[38] Erny D Dokalis N Mezö C Castoldi A Mossad O Staszewski O. Microbiota-derived acetate enables the metabolic fitness of the brain innate immune system during health and disease. Cell Metab. (2021) 33(11):2260–76.e7. 10.1016/j.cmet.2021.10.010 34731656

[39] Wenzel TJ Gates EJ Ranger AL Klegeris A. Short-chain fatty acids (SCFAs) alone or in combination regulate select immune functions of microglia-like cells. Mol Cell Neurosci. (2020) 105:103493. 10.1016/j.mcn.2020.103493 32333962

[40] Erny D Hrabě de Angelis AL Jaitin D Wieghofer P Staszewski O David E. Host microbiota constantly control maturation and function of microglia in the CNS. Nat Neurosci. (2015) 18(7):965–77. 10.1038/nn.4030 26030851 PMC5528863

[41] Kimura I Ichimura A Ohue-Kitano R Igarashi M. Free fatty acid receptors in health and disease. Physiol Rev. (2020) 100(1):171–210. 10.1152/physrev.00041.2018 31487233

[42] Lanza M Filippone A Ardizzone A Casili G Paterniti I Esposito E. SCFA treatment alleviates pathological signs of migraine and related intestinal alterations in a mouse model of NTG-induced migraine. Cells. (2021) 10(10):2756. 10.3390/cells10102756 34685736 PMC8535085

[43] Lanza M Filippone A Casili G Giuffrè L Scuderi SA Paterniti I. Supplementation with SCFAs re-establishes microbiota composition and attenuates hyperalgesia and pain in a mouse model of NTG-induced migraine. Int J Mol Sci. (2022) 23(9):4847. 10.3390/ijms23094847 35563235 PMC9100093

[44] Tang Y Guan L Li Z Li R Liu S Chen B. Synbiotics inhibits gut microbiome perturbation-induced prolongation of migraine-like pain by restoring short-chain fatty acid signaling. Neuropharmacology. (2026) 292:110914. 10.1016/j.neuropharm.2026.110914 41825506

[45] Tian N Yang F Zhao Y Jin M Sun F Liu M. Fecal amino acid and short-chain fatty acid profiles in children with migraine: a targeted metabolomics study. Front Microbiol. (2026) 17:1788386. 10.3389/fmicb.2026.1788386 42182020 PMC13195267

[46] Yano JM Yu K Donaldson GP Shastri GG Ann P Ma L. Indigenous bacteria from the gut microbiota regulate host serotonin biosynthesis. Cell. (2015) 161(2):264–76. 10.1016/j.cell.2015.02.047 25860609 PMC4393509

[47] Reigstad CS Salmonson CE Rainey JF 3rd Szurszewski JH Linden DR Sonnenburg JL. Gut microbes promote colonic serotonin production through an effect of short-chain fatty acids on enterochromaffin cells. FASEB J. (2015) 29(4):1395–403. 10.1096/fj.14-259598 25550456 PMC4396604

[48] Scott SA Fu J Chang PV. Microbial tryptophan metabolites regulate gut barrier function via the aryl hydrocarbon receptor. Proc Natl Acad Sci U S A. (2020) 117(32):19376–87. 10.1073/pnas.2000047117 32719140 PMC7431026

[49] Zelante T Iannitti RG Cunha C De Luca A Giovannini G Pieraccini G. Tryptophan catabolites from microbiota engage aryl hydrocarbon receptor and balance mucosal reactivity via interleukin-22. Immunity. (2013) 39(2):372–85. 10.1016/j.immuni.2013.08.003 23973224

[50] Rothhammer V Mascanfroni ID Bunse L Takenaka MC Kenison JE Mayo L. Type I interferons and microbial metabolites of tryptophan modulate astrocyte activity and central nervous system inflammation via the aryl hydrocarbon receptor. Nat Med. (2016) 22(6):586–97. 10.1038/nm.4106 27158906 PMC4899206

[51] Liu J Xi K Zhang L Han M Wang Q Liu X. Tryptophan metabolites and gut microbiota play an important role in pediatric migraine diagnosis. J Headache Pain. (2024) 25(1):2. 10.1186/s10194-023-01708-9 38177986 PMC10768287

[52] Luo T Xi Q Wang Q Wang Y Huang R Wang X. Exploratory metabolomic profiling in migraine with PFO reveals dysregulated pathways and highlights indoleacrylic acid. iScience. (2026) 29(5):115587. 10.1016/j.isci.2026.115587 42023154 PMC13098509

[53] Curto M Lionetto L Negro A Capi M Fazio F Giamberardino MA. Altered kynurenine pathway metabolites in serum of chronic migraine patients. J Headache Pain. (2016) 17:47. 10.1186/s10194-016-0638-5 PMC4851673 27130315

[54] Fila M Chojnacki J Pawlowska E Szczepanska J Chojnacki C Blasiak J. Kynurenine pathway of tryptophan metabolism in migraine and functional gastrointestinal disorders. Int J Mol Sci. (2021) 22(18):10134. 10.3390/ijms221810134 34576297 PMC8469852

[55] Greco R Demartini C Zanaboni AM Redavide E Pampalone S Toldi J. Effects of kynurenic acid analogue 1 (KYNA-A1) in nitroglycerin-induced hyperalgesia: targets and anti-migraine mechanisms. Cephalalgia. (2017) 37(13):1272–84. 10.1177/0333102416678000 27919017

[56] Fila M Chojnacki C Chojnacki J Blasiak J. The kynurenine pathway of tryptophan metabolism in abdominal migraine in children – a therapeutic potential? Eur J Paediatr Neurol. (2024) 48:1–12. 10.1016/j.ejpn.2023.11.001 37984006

[57] Whealy M Becker WJ. The 5-HT1B and 5-HT1D agonists in acute migraine therapy: ergotamine, dihydroergotamine, and the triptans. Handb Clin Neurol. (2024) 199:17–42. 10.1016/B978-0-12-823357-3.00008-2 38307644

[58] de Vries T Villalón CM MaassenVanDenBrink A. Pharmacological treatment of migraine: cGRP and 5-HT beyond the triptans. Pharmacol Ther. (2020) 211:107528. 10.1016/j.pharmthera.2020.107528 32173558

[59] Razeghi Jahromi S Togha M Ghorbani Z Hekmatdoost A Khorsha F Rafiee P. The association between dietary tryptophan intake and migraine. Neurol Sci. (2019) 40(11):2349–55. 10.1007/s10072-019-03984-3 31254181

[60] Rosier BT Johnston W Carda-Diéguez M Simpson A Cabello-Yeves E Piela K. Nitrate reduction capacity of the oral microbiota is impaired in periodontitis: potential implications for systemic nitric oxide availability. Int J Oral Sci. (2024) 16(1):1. 10.1038/s41368-023-00266-9 38177101 PMC10767001

[61] Vanhatalo A L'Heureux JE Black MI Blackwell JR Aizawa K Thompson C. Ageing modifies the oral microbiome, nitric oxide bioavailability and vascular responses to dietary nitrate supplementation. Free Radical Biol Med. (2025) 238:682–96. 10.1016/j.freeradbiomed.2025.07.002 40615058

[62] Gonzalez A Hyde E Sangwan N Gilbert JA Viirre E Knight R. Migraines are correlated with higher levels of nitrate-, nitrite-, and nitric oxide-reducing oral microbes in the American gut project cohort. mSystems. (2016) 1(5):e00105–16. 10.1128/mSystems.00105-16 27822557 PMC5080405

[63] Cho S Jung Y Oh H-S Yum J Song S Jeong JW. Oral and gut dysbiosis in migraine: oral microbial signatures as biomarkers of migraine. Neurol Neuroimmunol Neuroinflamm. (2025) 12(5):e200437. 10.1212/NXI.0000000000200437 40699951 PMC12221161

[64] Olesen J. Provocation of attacks to discover migraine signaling mechanisms and new drug targets: early history and future perspectives - a narrative review. J Headache Pain. (2024) 25(1):105. 10.1186/s10194-024-01796-1 38902612 PMC11188241

[65] He Y Shi Z Kashyap Y Messing RO Wang ZJ. Protein kinase cδ as a neuronal mechanism for headache in a chronic intermittent nitroglycerin model of migraine in mice. Pain. (2021) 162(10):2499–511. 10.1097/j.pain.0000000000002353 34108435 PMC8448952

[66] Demartini C Greco R Francavilla M Zanaboni AM Tassorelli C. Modelling migraine-related features in the nitroglycerin animal model: trigeminal hyperalgesia is associated with affective status and motor behavior. Physiol Behav. (2022) 256:113956. 10.1016/j.physbeh.2022.113956 36055415

[67] Ernstsen C Obelitz-Ryom K Kristensen DMB Olesen J Christensen SL Guo S. Mechanisms of GTN-induced migraine: role of NOS isoforms, sGC and peroxynitrite in a migraine relevant mouse model. Cephalalgia. (2024) 44(9):3331024241277542. 10.1177/03331024241277542 39314067

[68] Leclerc M Bedu-Ferrari C Etienne-Mesmin L Mariadassou M Lebreuilly L Tran S-L. Nitric oxide impacts human gut microbiota diversity and functionalities. mSystems. (2021) 6(5):e0055821. 10.1128/mSystems.00558-21 34519530 PMC8547463

[69] Matziouridou C Rocha SDC Haabeth OA Rudi K Carlsen H Kielland A. iNOS- and NOX1-dependent ROS production maintains bacterial homeostasis in the ileum of mice. Mucosal Immunol. (2018) 11(3):774–84. 10.1038/mi.2017.106 29210363

[70] Kang L Tang W Zhang Y Zhang M Liu J Li Y. The gut microbiome modulates nitroglycerin-induced migraine-related hyperalgesia in mice. Cephalalgia. (2022) 42(6):490–9. 10.1177/03331024211050036 34644194

[71] Moran SP Rosier BT Henriquez FL Burleigh MC. The effects of nitrate on the oral microbiome: a systematic review investigating prebiotic potential. J Oral Microbiol. (2024) 16(1):2322228. 10.1080/20002297.2024.2322228 38420038 PMC10901185

[72] Fiorani M Del Vecchio LE Dargenio P Kaitsas F Rozera T Porcari S. Histamine-producing bacteria and their role in gastrointestinal disorders. Expert Rev Gastroenterol Hepatol. (2023) 17(7):709–18. 10.1080/17474124.2023.2230865 37394958

[73] Öz Tunçer G Akbaş Y Köker A Aydın Köker S Tural Kara T Çoban Y. Serum zonulin levels in pediatric migraine. Pediatr Neurol. (2023) 144:80–3. 10.1016/j.pediatrneurol.2023.04.014 37196600

[74] Della Pietra A Russo AF. Overlapping pathways of migraine and the endocannabinoid system: potential therapeutic targets. Neurotherapeutics. (2026) 23(3):e00833. 10.1016/j.neurot.2026.e00833 41549028 PMC12996789

[75] Greco R Demartini C Zanaboni AM Tumelero E De Icco R Sances G. Peripheral changes of endocannabinoid system components in episodic and chronic migraine patients: a pilot study. Cephalalgia. (2021) 41(2):185–96. 10.1177/0333102420949201 32967434

[76] Fornelos N Franzosa EA Bishai J Annand JW Oka A Lloyd-Price J. Growth effects of N-acylethanolamines on gut bacteria reflect altered bacterial abundances in inflammatory bowel disease. Nat Microbiol. (2020) 5(3):486–97. 10.1038/s41564-019-0655-7 31959971 PMC7047597

[77] Kumari N Kumari R Dua A Singh M Kumar R Singh P. From gut to hormones: unraveling the role of gut microbiota in (phyto)estrogen modulation in health and disease. Mol Nutr Food Res. (2024) 68(6):e2300688. 10.1002/mnfr.202300688 38342595

[78] Hu S Ding Q Zhang W Kang M Ma J Zhao L. Gut microbial beta-glucuronidase: a vital regulator in female estrogen metabolism. Gut Microbes. (2023) 15(1):2236749. 10.1080/19490976.2023.2236749 37559394 PMC10416750

[79] Ervin SM Li H Lim L Roberts LR Liang X Mani S. Gut microbial β-glucuronidases reactivate estrogens as components of the estrobolome that reactivate estrogens. J Biol Chem. (2019) 294(49):18586–99. 10.1074/jbc.RA119.010950 31636122 PMC6901331

[80] Krause DN Warfvinge K Haanes KA Edvinsson L. Hormonal influences in migraine - interactions of oestrogen, oxytocin and CGRP. Nat Rev Neurol. (2021) 17(10):621–33. 10.1038/s41582-021-00544-2 34545218

[81] Li VW Dong TS Funes D Hernandez L Kushnir NR Nair D. Mass spectrometric profiling of primary estrogens and estrogen metabolites in human stool and plasma partially elucidates the role of the gut microbiome in estrogen recycling. Mol Cell Endocrinol. (2025) 603:112534. 10.1016/j.mce.2025.112534 40180172 PMC13006107

[82] de Mora F Messlinger K. Is calcitonin gene-related peptide (CGRP) the missing link in food histamine-induced migraine? A review of functional gut-to-trigeminovascular system connections. Drug Discov Today. (2024) 29(4):103941. 10.1016/j.drudis.2024.103941 38447930

[83] Olga Grodzka I. Gut microbiota, probiotics, and migraine: a clinical review and meta-analysis. J Oral Facial Pain Headache. (2025) 39(3):13–26. 10.22514/jofph.2025.043 41070562 PMC12520441

[84] Ghavami A Khorvash F Heidari Z Khalesi S Askari G. Effect of synbiotic supplementation on migraine characteristics and inflammatory biomarkers in women with migraine: results of a randomized controlled trial. Pharmacol Res. (2021) 169:105668. 10.1016/j.phrs.2021.105668 33989763

[85] Roldán-Ruiz A Bertotti G López-Moreno M. Effects of dietary interventions in patients with migraine: a systematic review. Nutr Rev. (2025) 83(7):e1815–27. 10.1093/nutrit/nuae188 39749874

[86] Liu J Liu H Li W Huang S. Association between dietary index for gut microbiota and self-reported severe headache or migraine in U.S. adults: a cross-sectional study from NHANES. Front Nutr. (2025) 12:1549251. 10.3389/fnut.2025.1549251 40230722 PMC11994445

[87] Kappéter Á Sipos D Varga A Vigvári S Halda-Kiss B Péterfi Z. Migraine as a disease associated with dysbiosis and possible therapy with fecal microbiota transplantation. Microorganisms. (2023) 11(8):2083. 10.3390/microorganisms11082083 PMC10458656 37630643

[88] Tirani SA Saneei P Khorvash F Askari G. Effects of probiotic and vitamin D co-supplementation on migraine index, quality of life, and oxidative stress in adults with migraine headache: a randomized triple-blinded clinical trial. Eur J Nutr. (2025) 64(4):179. 10.1007/s00394-025-03687-w 40360751

